# *N*-Benzoyl-*N*′,*N*′′-dicyclo­hexyl­phospho­ric triamide

**DOI:** 10.1107/S1600536811016679

**Published:** 2011-05-07

**Authors:** Mehrdad Pourayoubi, Mahnaz Rostami Chaijan, Laura Torre-Fernández, Santiago García-Granda

**Affiliations:** a Department of Chemistry, Ferdowsi University of Mashhad, Mashhad 91779, Iran; b Departamento de Química Física y Analítica, Facultad de Química, Universidad de Oviedo–CINN, C/ Julián Clavería, 8, 33006 Oviedo, Asturias, Spain

## Abstract

In the title compound, C_19_H_30_N_3_O_2_P, the central P atom has a distorted tetra­hedral configuration. The N atoms in both cyclo­hexyl­amide moieties exhibit a slight deviation [0.32 (7) and 0.44 (6) Å] from planarity, while the benzoyl­amide N atom is planar [0.11 (3) Å]. In the crystal, mol­ecules are linked *via* N—H⋯O(P) and N—H⋯O(C) hydrogen bonds, forming *R*_2_^2^(10) rings within linear arrangements parallel to the *b* axis.

## Related literature

For the synthesis and a spectroscopic study of title compound, see: Gholivand *et al.* (2006 ▶). For bond lengths in related structures, see: Sabbaghi *et al.* (2010 ▶); Rudd *et al.* (1996 ▶). For hydrogen-bond motifs, see: Etter *et al.* (1990 ▶); Bernstein *et al.* (1995 ▶).
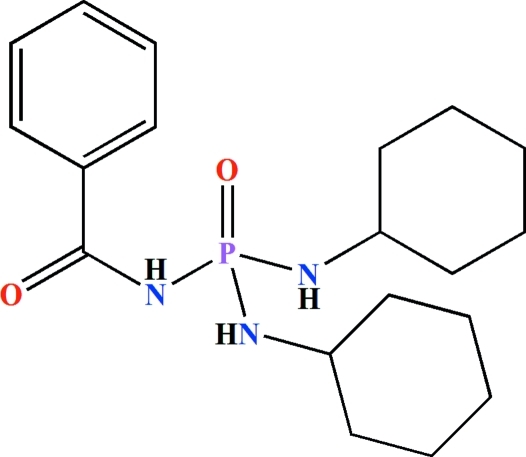


## Experimental

### 

#### Crystal data

C_19_H_30_N_3_O_2_P
*M* *_r_* = 363.43
Monoclinic, 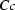

*a* = 20.9904 (17) Å
*b* = 5.1503 (2) Å
*c* = 21.1125 (18) Å
β = 121.955 (11)°
*V* = 1936.5 (2) Å^3^
*Z* = 4
Cu *K*α radiation
μ = 1.39 mm^−1^
*T* = 293 K
0.28 × 0.05 × 0.01 mm

#### Data collection

Oxford Diffraction Xcalibur Ruby Gemini diffractometer
Absorption correction: multi-scan (*CrysAlis PRO*; Oxford Diffraction, 2010 ▶) *T*_min_ = 0.978, *T*_max_ = 1.000
2758 measured reflections
2758 independent reflections
2294 reflections with *I* > 2σ(*I*)
*R*_int_ = 0.064

#### Refinement

*R*[*F* ^2^ > 2σ(*F* ^2^)] = 0.051
*wR*(*F* ^2^) = 0.122
*S* = 1.05
2758 reflections
239 parameters
2 restraints
H atoms treated by a mixture of independent and constrained refinement
Δρ_max_ = 0.46 e Å^−3^
Δρ_min_ = −0.18 e Å^−3^
Absolute structure: Flack (1983 ▶), 908 Friedel pairs
Flack parameter: 0.11 (4)

### 

Data collection: *CrysAlis PRO* (Oxford Diffraction, 2010 ▶); cell refinement: *CrysAlis PRO*; data reduction: *CrysAlis PRO*; program(s) used to solve structure: *SUPERFLIP* (Palatinus & Chapuis, 2007 ▶); program(s) used to refine structure: *SHELXL97* (Sheldrick, 2008 ▶); molecular graphics: *Mercury* (Macrae *et al.*, 2008 ▶); software used to prepare material for publication: *WinGX* (Farrugia, 1999 ▶).

## Supplementary Material

Crystal structure: contains datablocks global, I. DOI: 10.1107/S1600536811016679/ld2010sup1.cif

Structure factors: contains datablocks I. DOI: 10.1107/S1600536811016679/ld2010Isup2.hkl

Additional supplementary materials:  crystallographic information; 3D view; checkCIF report

## Figures and Tables

**Table 1:** Hydrogen-bond geometry (Å, °)

*D*—H⋯*A*	*D*—H	H⋯*A*	*D*⋯*A*	*D*—H⋯*A*
N4—H4⋯O3^i^	0.90 (4)	2.16 (4)	2.988 (4)	154 (3)
N5—H5⋯O2^ii^	0.77 (5)	2.30 (6)	3.019 (5)	156 (6)

Symmetry codes: (i) 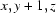; (ii) 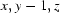.

**Fig. 1.**
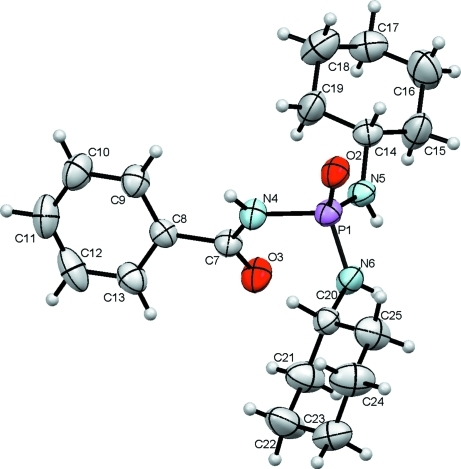
An ORTEP-style plot of title compound with labeling. Ellipsoids are given at the 50% probability level.

**Fig. 2.**
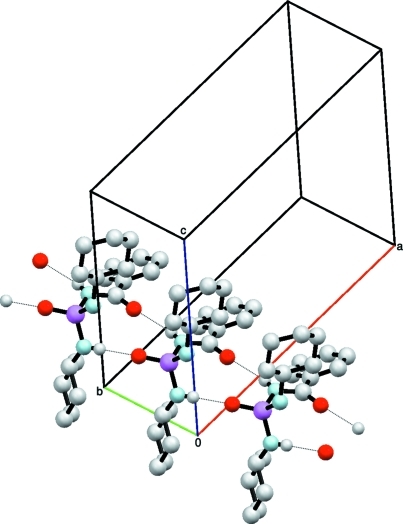
Partial packing view showing the formation of the chain through N—H···O hydrogen bonds (along the b axis) which are shown as dotted lines. H atoms not involved in the hydrogen bondings are omitted.

## supplementary crystallographic information

### Comment

The synthesis of the title compound, C_6_H_5_C(O)NHP(O)[NHC_6_H_11_]_2_, was previously published by Gholivand *et al.* (2006). Here, we report its crystal structure (Fig. 1).

The P═O, C═O and P—N bond lengths match those found for other compounds with the [(N)(N)P(O)NHC(O)] skeleton (Sabbaghi *et al.*, 2010). The nitrogen atoms show *sp*^2^ character and the environment of N atom in C(O)NHP(O) moiety is practically planar. The tetrahedral configuration of phosphorus atom is significantly distorted (Fig. 1) as it has been noted for other phosphoric triamides and their chalco-derivatives (Rudd *et al.*, 1996): the bond angles at the P atom vary in the range from 102.7 (2)° to 117.2 (2)°, while the P–N bond distances range form 1.615 (4) to 1.730 (4) Å. Cyclohexyl groups are in a chair conformation with the adjacent NH groups oriented equatorially.

The NH unit of the C(O)NHP(O) moiety adopts a *syn* orientation with respect to the phosphoryl group; moreover, the NH units of two C_6_H_11_NH moieties are in a *syn* orientation with respect to each other.

In the crystal structure, the molecules are linked *via* N—H···O═P and N—H···O═C hydrogen bonds, in which carbonyl oxygen interacts with benzamide N—H and P(O) group binds to a cyclohexylamido moiety. This way, *R_2_^2^*(10) rings are built (Etter *et al.*, 1990; Bernstein *et al.*, 1995), that are further connected in linear arrangements along *y* axis (Table 1, Fig. 2).

### Experimental

C_6_H_5_C(O)NHP(O)[NHC_6_H_11_]_2_ was prepared according to the procedure reported by Gholivand *et al.* (2006). Single crystals of title compound were obtained from CH_3_OH after slow evaporation at room temperature.

### Refinement

At the end of the refinement the highest peak in the electron density was 0.460 e Å ^-3^, while the deepest hole was -0.180 e Å ^-3^.

### Crystal data

**Table:**

C_19_H_30_N_3_O_2_P	*F*(000) = 784
*M**_r_* = 363.43	*D*_x_ = 1.247 Mg m^−^^3^
Monoclinic, *C**c*	Cu *K*α radiation, λ = 1.54180 Å
Hall symbol: C -2yc	Cell parameters from 1733 reflections
*a* = 20.9904 (17) Å	θ = 4.2–70.2°
*b* = 5.1503 (2) Å	µ = 1.39 mm^−^^1^
*c* = 21.1125 (18) Å	*T* = 293 K
β = 121.955 (11)°	Prismatic, colorless
*V* = 1936.5 (2) Å^3^	0.28 × 0.05 × 0.01 mm
*Z* = 4	

### Data collection

**Table:**

Oxford Diffraction Xcalibur Ruby Gemini diffractometer	2758 independent reflections
Radiation source: Enhance (Cu) X-ray Source	2294 reflections with *I* > 2σ(*I*)
graphite	*R*_int_ = 0.064
Detector resolution: 10.2673 pixels mm^-1^	θ_max_ = 70.3°, θ_min_ = 4.8°
ω scans	*h* = −24→24
Absorption correction: multi-scan (*CrysAlis PRO*; Oxford Diffraction, 2010)	*k* = 0→6
*T*_min_ = 0.978, *T*_max_ = 1.000	*l* = −25→25
2758 measured reflections	

### Refinement

**Table:**

Refinement on *F*^2^	Secondary atom site location: difference Fourier map
Least-squares matrix: full	Hydrogen site location: inferred from neighbouring sites
*R*[*F*^2^ > 2σ(*F*^2^)] = 0.051	H atoms treated by a mixture of independent and constrained refinement
*wR*(*F*^2^) = 0.122	*w* = 1/[σ^2^(*F*_o_^2^) + (0.0489*P*)^2^] where *P* = (*F*_o_^2^ + 2*F*_c_^2^)/3
*S* = 1.05	(Δ/σ)_max_ < 0.001
2758 reflections	Δρ_max_ = 0.46 e Å^−^^3^
239 parameters	Δρ_min_ = −0.18 e Å^−^^3^
2 restraints	Absolute structure: Flack (1983), 908 Friedel pairs
Primary atom site location: structure-invariant direct methods	Flack parameter: 0.11 (4)

### Special details

**Table:**

Geometry. All e.s.d.'s (except the e.s.d. in the dihedral angle between two l.s. planes) are estimated using the full covariance matrix. The cell e.s.d.'s are taken into account individually in the estimation of e.s.d.'s in distances, angles and torsion angles; correlations between e.s.d.'s in cell parameters are only used when they are defined by crystal symmetry. An approximate (isotropic) treatment of cell e.s.d.'s is used for estimating e.s.d.'s involving l.s. planes.
Refinement. Refinement of *F*^2^ against ALL reflections. The weighted *R*-factor *wR* and goodness of fit *S* are based on *F*^2^, conventional *R*-factors *R* are based on *F*, with *F* set to zero for negative *F*^2^. The threshold expression of *F*^2^ > σ(*F*^2^) is used only for calculating *R*-factors(gt) *etc*. and is not relevant to the choice of reflections for refinement. *R*-factors based on *F*^2^ are statistically about twice as large as those based on *F*, and *R*- factors based on ALL data will be even larger.

### Fractional atomic coordinates and isotropic or equivalent isotropic displacement parameters (Å^2^)

**Table:**

	*x*	*y*	*z*	*U*_iso_*/*U*_eq_	
P1	0.00072 (6)	0.2629 (2)	0.30701 (6)	0.0403 (2)	
O2	−0.02706 (18)	0.5292 (5)	0.30029 (19)	0.0504 (8)	
O3	0.11281 (18)	−0.1251 (6)	0.3206 (2)	0.0578 (9)	
N4	0.0865 (2)	0.3029 (6)	0.3150 (2)	0.0435 (8)	
N5	−0.0520 (2)	0.0724 (7)	0.2376 (2)	0.0438 (9)	
N6	0.0150 (2)	0.0959 (8)	0.3781 (2)	0.0458 (9)	
C7	0.1289 (2)	0.1008 (8)	0.3169 (2)	0.0398 (9)	
C8	0.1958 (2)	0.1615 (7)	0.3117 (2)	0.0401 (9)	
C9	0.1990 (3)	0.3714 (9)	0.2727 (3)	0.0490 (11)	
H9	0.1598	0.4905	0.2518	0.059*	
C10	0.2585 (3)	0.4083 (11)	0.2640 (3)	0.0616 (13)	
H10	0.2590	0.5492	0.2367	0.074*	
C11	0.3180 (3)	0.2347 (12)	0.2961 (3)	0.0677 (14)	
H11	0.3587	0.2600	0.2907	0.081*	
C12	0.3169 (3)	0.0239 (12)	0.3362 (3)	0.0722 (16)	
H12	0.3569	−0.0923	0.3577	0.087*	
C13	0.2559 (3)	−0.0130 (10)	0.3440 (3)	0.0533 (12)	
H13	0.2549	−0.1546	0.3708	0.064*	
C14	−0.0945 (2)	0.1484 (9)	0.1595 (2)	0.0451 (10)	
H14	−0.1203	0.3120	0.1554	0.054*	
C15	−0.1535 (3)	−0.0560 (13)	0.1154 (3)	0.0727 (17)	
H15A	−0.1872	−0.0665	0.1336	0.087*	
H15B	−0.1292	−0.2234	0.1235	0.087*	
C16	−0.1994 (3)	0.0014 (16)	0.0314 (3)	0.0846 (19)	
H16A	−0.2337	−0.1413	0.0055	0.102*	
H16B	−0.2290	0.1571	0.0225	0.102*	
C17	−0.1502 (3)	0.0381 (11)	0.0011 (3)	0.0638 (13)	
H17B	−0.1806	0.0862	−0.0511	0.077*	
H17A	−0.1251	−0.1241	0.0045	0.077*	
C18	−0.0922 (4)	0.2463 (12)	0.0436 (4)	0.0774 (17)	
H18A	−0.1172	0.4125	0.0352	0.093*	
H18B	−0.0589	0.2579	0.0250	0.093*	
C19	−0.0464 (3)	0.1896 (13)	0.1269 (3)	0.0683 (16)	
H19A	−0.0162	0.0356	0.1356	0.082*	
H19B	−0.0124	0.3335	0.1525	0.082*	
C20	0.0677 (2)	0.1637 (8)	0.4557 (2)	0.0426 (9)	
H20	0.0962	0.3151	0.4562	0.051*	
C21	0.1229 (3)	−0.0486 (12)	0.4978 (3)	0.0685 (15)	
H21A	0.1533	−0.0758	0.4763	0.082*	
H21B	0.0956	−0.2081	0.4916	0.082*	
C22	0.1746 (3)	0.0067 (15)	0.5810 (3)	0.0805 (19)	
H22A	0.2054	−0.1448	0.6055	0.097*	
H22B	0.2077	0.1498	0.5878	0.097*	
C23	0.1303 (3)	0.0742 (10)	0.6167 (3)	0.0650 (14)	
H23B	0.1019	−0.0762	0.6155	0.078*	
H23A	0.1646	0.1218	0.6685	0.078*	
C24	0.0777 (4)	0.2951 (13)	0.5762 (3)	0.0771 (18)	
H24A	0.1068	0.4505	0.5829	0.093*	
H24B	0.0477	0.3271	0.5979	0.093*	
C25	0.0261 (3)	0.2433 (12)	0.4937 (3)	0.0654 (15)	
H25A	−0.0029	0.3987	0.4697	0.078*	
H25B	−0.0088	0.1066	0.4868	0.078*	
H4	0.1046 (19)	0.457 (7)	0.3124 (19)	0.015 (8)*	
H6	0.014 (3)	−0.052 (9)	0.371 (3)	0.037 (13)*	
H5	−0.038 (3)	−0.067 (11)	0.248 (3)	0.062 (17)*	

### Atomic displacement parameters (Å^2^)

**Table:**

	*U*^11^	*U*^22^	*U*^33^	*U*^12^	*U*^13^	*U*^23^
P1	0.0447 (5)	0.0336 (4)	0.0472 (5)	0.0016 (5)	0.0276 (4)	0.0017 (5)
O2	0.0546 (17)	0.0351 (15)	0.071 (2)	0.0019 (13)	0.0401 (17)	0.0037 (14)
O3	0.061 (2)	0.0334 (17)	0.089 (3)	0.0009 (14)	0.047 (2)	0.0017 (15)
N4	0.0453 (19)	0.0326 (18)	0.060 (2)	0.0006 (15)	0.0333 (18)	−0.0008 (15)
N5	0.048 (2)	0.035 (2)	0.047 (2)	0.0003 (16)	0.0244 (18)	0.0047 (16)
N6	0.054 (2)	0.044 (2)	0.046 (2)	−0.0048 (18)	0.0301 (19)	−0.0002 (17)
C7	0.0389 (19)	0.036 (2)	0.045 (2)	0.0004 (17)	0.0228 (18)	0.0000 (17)
C8	0.041 (2)	0.0367 (19)	0.042 (2)	0.0018 (17)	0.0213 (18)	−0.0041 (17)
C9	0.052 (3)	0.045 (2)	0.059 (3)	0.002 (2)	0.035 (2)	−0.003 (2)
C10	0.068 (3)	0.062 (3)	0.076 (3)	−0.003 (3)	0.053 (3)	−0.003 (3)
C11	0.055 (3)	0.074 (4)	0.091 (4)	−0.011 (3)	0.050 (3)	−0.019 (3)
C12	0.037 (3)	0.076 (4)	0.091 (4)	0.011 (2)	0.026 (3)	−0.016 (3)
C13	0.046 (2)	0.049 (3)	0.057 (3)	0.006 (2)	0.022 (2)	−0.002 (2)
C14	0.048 (2)	0.042 (2)	0.045 (3)	0.0027 (19)	0.024 (2)	0.0002 (19)
C15	0.064 (3)	0.103 (5)	0.050 (3)	−0.034 (3)	0.030 (3)	−0.006 (3)
C16	0.059 (3)	0.116 (5)	0.064 (4)	−0.015 (3)	0.023 (3)	−0.008 (4)
C17	0.079 (3)	0.057 (3)	0.052 (3)	0.003 (3)	0.032 (3)	0.004 (2)
C18	0.107 (4)	0.070 (4)	0.067 (4)	−0.019 (4)	0.054 (4)	−0.003 (3)
C19	0.060 (3)	0.091 (4)	0.056 (3)	−0.024 (3)	0.033 (3)	0.001 (3)
C20	0.045 (2)	0.041 (2)	0.045 (2)	0.0004 (18)	0.025 (2)	0.0032 (18)
C21	0.074 (3)	0.077 (4)	0.057 (3)	0.027 (3)	0.037 (3)	0.010 (3)
C22	0.065 (3)	0.114 (5)	0.051 (4)	0.028 (4)	0.022 (3)	0.018 (3)
C23	0.083 (4)	0.055 (3)	0.053 (3)	−0.001 (3)	0.034 (3)	0.004 (2)
C24	0.102 (5)	0.075 (4)	0.056 (3)	0.017 (4)	0.043 (3)	0.000 (3)
C25	0.071 (3)	0.078 (4)	0.057 (3)	0.023 (3)	0.040 (3)	0.006 (3)

### Geometric parameters (Å, °)

**Table:**

P1—O2	1.468 (3)	C16—C17	1.487 (8)
P1—N6	1.615 (4)	C16—H16A	0.9700
P1—N5	1.618 (4)	C16—H16B	0.9700
P1—N4	1.730 (4)	C17—C18	1.509 (8)
O3—C7	1.225 (5)	C17—H17B	0.9700
N4—C7	1.356 (5)	C17—H17A	0.9700
N4—H4	0.90 (4)	C18—C19	1.521 (9)
N5—C14	1.454 (6)	C18—H18A	0.9700
N5—H5	0.77 (5)	C18—H18B	0.9700
N6—C20	1.453 (6)	C19—H19A	0.9700
N6—H6	0.78 (4)	C19—H19B	0.9700
C7—C8	1.499 (6)	C20—C21	1.496 (7)
C8—C9	1.382 (6)	C20—C25	1.521 (6)
C8—C13	1.397 (6)	C20—H20	0.9800
C9—C10	1.366 (7)	C21—C22	1.525 (8)
C9—H9	0.9300	C21—H21A	0.9700
C10—C11	1.387 (8)	C21—H21B	0.9700
C10—H10	0.9300	C22—C23	1.514 (8)
C11—C12	1.383 (9)	C22—H22A	0.9700
C11—H11	0.9300	C22—H22B	0.9700
C12—C13	1.389 (7)	C23—C24	1.498 (8)
C12—H12	0.9300	C23—H23B	0.9700
C13—H13	0.9300	C23—H23A	0.9700
C14—C19	1.507 (7)	C24—C25	1.511 (8)
C14—C15	1.513 (7)	C24—H24A	0.9700
C14—H14	0.9800	C24—H24B	0.9700
C15—C16	1.533 (8)	C25—H25A	0.9700
C15—H15A	0.9700	C25—H25B	0.9700
C15—H15B	0.9700		
			
O2—P1—N6	117.2 (2)	C16—C17—C18	111.1 (5)
O2—P1—N5	115.7 (2)	C16—C17—H17B	109.4
N6—P1—N5	102.73 (19)	C18—C17—H17B	109.4
O2—P1—N4	103.77 (18)	C16—C17—H17A	109.4
N6—P1—N4	107.5 (2)	C18—C17—H17A	109.4
N5—P1—N4	109.79 (19)	H17B—C17—H17A	108.0
C7—N4—P1	123.0 (3)	C17—C18—C19	111.6 (5)
C7—N4—H4	113 (2)	C17—C18—H18A	109.3
P1—N4—H4	124 (2)	C19—C18—H18A	109.3
C14—N5—P1	125.4 (3)	C17—C18—H18B	109.3
C14—N5—H5	120 (4)	C19—C18—H18B	109.3
P1—N5—H5	109 (4)	H18A—C18—H18B	108.0
C20—N6—P1	125.2 (3)	C14—C19—C18	112.8 (5)
C20—N6—H6	113 (4)	C14—C19—H19A	109.0
P1—N6—H6	112 (4)	C18—C19—H19A	109.0
O3—C7—N4	122.2 (4)	C14—C19—H19B	109.0
O3—C7—C8	120.2 (4)	C18—C19—H19B	109.0
N4—C7—C8	117.6 (3)	H19A—C19—H19B	107.8
C9—C8—C13	118.6 (4)	N6—C20—C21	112.4 (4)
C9—C8—C7	123.4 (4)	N6—C20—C25	110.6 (4)
C13—C8—C7	117.9 (4)	C21—C20—C25	111.4 (4)
C10—C9—C8	121.6 (5)	N6—C20—H20	107.4
C10—C9—H9	119.2	C21—C20—H20	107.4
C8—C9—H9	119.2	C25—C20—H20	107.4
C9—C10—C11	119.7 (5)	C20—C21—C22	113.7 (5)
C9—C10—H10	120.1	C20—C21—H21A	108.8
C11—C10—H10	120.1	C22—C21—H21A	108.8
C12—C11—C10	120.2 (5)	C20—C21—H21B	108.8
C12—C11—H11	119.9	C22—C21—H21B	108.8
C10—C11—H11	119.9	H21A—C21—H21B	107.7
C11—C12—C13	119.6 (5)	C23—C22—C21	111.5 (5)
C11—C12—H12	120.2	C23—C22—H22A	109.3
C13—C12—H12	120.2	C21—C22—H22A	109.3
C12—C13—C8	120.3 (5)	C23—C22—H22B	109.3
C12—C13—H13	119.9	C21—C22—H22B	109.3
C8—C13—H13	119.9	H22A—C22—H22B	108.0
N5—C14—C19	113.5 (4)	C24—C23—C22	110.6 (5)
N5—C14—C15	108.8 (4)	C24—C23—H23B	109.5
C19—C14—C15	110.2 (4)	C22—C23—H23B	109.5
N5—C14—H14	108.1	C24—C23—H23A	109.5
C19—C14—H14	108.1	C22—C23—H23A	109.5
C15—C14—H14	108.1	H23B—C23—H23A	108.1
C14—C15—C16	112.7 (5)	C23—C24—C25	112.7 (5)
C14—C15—H15A	109.1	C23—C24—H24A	109.1
C16—C15—H15A	109.1	C25—C24—H24A	109.1
C14—C15—H15B	109.1	C23—C24—H24B	109.1
C16—C15—H15B	109.1	C25—C24—H24B	109.1
H15A—C15—H15B	107.8	H24A—C24—H24B	107.8
C17—C16—C15	111.6 (5)	C24—C25—C20	113.2 (5)
C17—C16—H16A	109.3	C24—C25—H25A	108.9
C15—C16—H16A	109.3	C20—C25—H25A	108.9
C17—C16—H16B	109.3	C24—C25—H25B	108.9
C15—C16—H16B	109.3	C20—C25—H25B	108.9
H16A—C16—H16B	108.0	H25A—C25—H25B	107.8
			
O2—P1—N4—C7	−174.9 (4)	C7—C8—C13—C12	175.4 (4)
N6—P1—N4—C7	60.3 (4)	P1—N5—C14—C19	74.0 (5)
N5—P1—N4—C7	−50.7 (4)	P1—N5—C14—C15	−162.9 (4)
O2—P1—N5—C14	37.2 (4)	N5—C14—C15—C16	−177.7 (5)
N6—P1—N5—C14	166.0 (4)	C19—C14—C15—C16	−52.6 (7)
N4—P1—N5—C14	−79.8 (4)	C14—C15—C16—C17	54.8 (8)
O2—P1—N6—C20	−61.0 (4)	C15—C16—C17—C18	−55.0 (8)
N5—P1—N6—C20	171.1 (4)	C16—C17—C18—C19	55.1 (8)
N4—P1—N6—C20	55.2 (4)	N5—C14—C19—C18	175.1 (5)
P1—N4—C7—O3	−7.5 (6)	C15—C14—C19—C18	52.8 (7)
P1—N4—C7—C8	170.5 (3)	C17—C18—C19—C14	−54.7 (8)
O3—C7—C8—C9	147.1 (4)	P1—N6—C20—C21	−124.3 (4)
N4—C7—C8—C9	−31.0 (6)	P1—N6—C20—C25	110.5 (4)
O3—C7—C8—C13	−28.7 (6)	N6—C20—C21—C22	−174.9 (5)
N4—C7—C8—C13	153.3 (4)	C25—C20—C21—C22	−50.1 (7)
C13—C8—C9—C10	1.2 (7)	C20—C21—C22—C23	53.6 (7)
C7—C8—C9—C10	−174.5 (5)	C21—C22—C23—C24	−54.7 (7)
C8—C9—C10—C11	−1.2 (8)	C22—C23—C24—C25	55.0 (7)
C9—C10—C11—C12	0.5 (8)	C23—C24—C25—C20	−52.9 (7)
C10—C11—C12—C13	0.1 (8)	N6—C20—C25—C24	175.3 (5)
C11—C12—C13—C8	−0.1 (8)	C21—C20—C25—C24	49.5 (7)
C9—C8—C13—C12	−0.6 (7)		

### Hydrogen-bond geometry (Å, °)

**Table:**

*D*—H···*A*	*D*—H	H···*A*	*D*···*A*	*D*—H···*A*
N4—H4···O3^i^	0.90 (4)	2.16 (4)	2.988 (4)	154 (3)
N5—H5···O2^ii^	0.77 (5)	2.30 (6)	3.019 (5)	156 (6)

Symmetry codes: (i) *x*, *y*+1, *z*; (ii) *x*, *y*−1, *z*.
